# Corrosion Behavior of Ni/NiCr/NiCrAlSi Composite Coating on Copper for Application as a Heat Exchanger in Sea Water

**DOI:** 10.3390/nano13243129

**Published:** 2023-12-13

**Authors:** Hao Du, Jiayuan Wen, Guihong Song, Hao Wu, Yansheng Yin

**Affiliations:** 1 Guangdong Key Laboratory of Materials and Equipment in Harsh Marine Environment, Guangzhou Maritime University, Guangzhou 510725, China; duhao@gzmtu.edu.cn (H.D.); ysyin@shmtu.edu.cn (Y.Y.); 2 School of Naval Architecture and Ocean Engineering, Guangzhou Maritime University, Guangzhou 510725, China; 3 School of Material Science and Technology, Shenyang University of Technology, Shenyang 110870, China; wenjy@sut.edu.cn

**Keywords:** composite coating, corrosion behavior, magnetron sputtering

## Abstract

This study introduces a novel Ni/NiCr/NiCrAlSi composite coating to enhance the corrosion resistance of copper, particularly for its use in marine heat exchangers. Utilizing characterization techniques such as scanning electron microscopy (SEM), energy dispersive spectroscopy (EDS), X-ray diffraction (XRD), potentiodynamic polarization, and electrochemical impedance spectroscopy (EIS), the paper investigates the coating’s composition, structure, and corrosion resistance in 3.5 wt.% NaCl aqueous solutions. A significant focus is placed on the role of aluminum within the NiCrAlSi layer, examining its influence on the coating’s structure and corrosion behavior. The results indicate that the NiCrAlSi layer with an aluminum content of 5.49 at.% exhibits the most improved corrosion resistance, characterized by the highest corrosion potential and a corrosion current density that is more than one order of magnitude lower compared to the Ni/NiCr coating. The effectiveness of this composite coating is attributed to its multilayer structure and the synergistic effect of alloying elements Cr, Al, and Si, which collectively inhibit corrosive medium penetration. These insights present the Ni/NiCr/NiCrAlSi coating as a promising candidate for copper protection in sea water environments, merging enhanced durability with cost-effectiveness.

## 1. Introduction

Copper exhibits superior thermal conductivity, high malleability, ease of joining, and excellent recyclability, making it an outstanding material for use in heat exchangers across various applications [1,2,3]. However, its application in marine environments is limited due to its poor corrosion resistance in sea water. While atmospheric exposure can lead to the formation of a self-protective copper oxide film, enhancing corrosion resistance, this film is compromised in sea water due to the penetration of chloride ions (Cl^−^), which leads to significant corrosion [4,5,6]. Additionally, the resultant corrosion products may detach, leading to the degradation of the copper material and potential failure of the equipment, either temporarily or permanently [7].

Different methods have been reported to improve the corrosion resistance of copper, such as alloying, composites, and employing coatings, inhibitors, and others. Among these methods, although alloying elements can improve the corrosion resistance and mechanical strength of copper, it will generally mean the electrical and thermal conductivity of copper [8] are sacrificed, making it unacceptable for application as a heat exchanger. Some researchers have reported that metallic sandwich composites, such as Cu/Al [9], Cu/Al/Cu [10], and Ni/Cu/Ni [11], combine the advantages of both copper and aluminum or nickel, and that not only the corrosion resistance but also the strength of copper are improved by the other element. It is interesting to find that this method may deal with the problem of keeping the thermal conductivity of copper for high-efficiency heat dissipation [12]; however, it is more useful for applications in atmosphere environments, not in sea water [13]. As a new corrosion inhibition technology [14], constructing a superhydrophobic surface which can also be obtained through films or coatings [15] may greatly improve the corrosion resistance of copper in sea water. It should be mentioned that superhydrophobic surfaces generally show good corrosion protection in a short time [16], but how to deal with the stability and the durability related to corrosion resistance is still a challenge. To protect the environment, people have opted for the application of films or coatings for improving the corrosion resistance of copper [2,17]. Among the available deposition methods, physical vapor deposition (PVD) is more attractive for preparing protective coatings on copper for improved corrosion resistance. In addition, metallic targets used to fabricate coatings by the PVD process can be recycled, providing an environmentally friendly management solution. Out of the available PVD methods, magnetron sputtering (MS) exhibits the “rapid quenching effect” with a high cooling rate which can restrict the diffusion of elements and thus restrain the nucleation and growth of intermetallic compounds, in favor of the formation of a film with several elements. Therefore, more homogeneous, multicomponent alloy coatings (3, 4, and even more elements) with a dense microstructure and defined chemical composition and properties can be obtained, which is very important especially in applications related to corrosion resistance. Furthermore, MS allows greater control of process parameters in order to adjust the microstructure, chemical composition, thickness, and properties of the coatings according to the required application.

MS films or coatings are more promising for protecting copper with regard to application in sea water. However, growth defects (pores, pinholes, inter-columnar voids, grain boundaries) are inevitable in the deposition and may compromise the effectiveness of the obtained films or coatings with regard to corrosion resistance. These defects not only provide “channels” for the permeation of corrosive medium to the substrate [18,19] but also are the “weak” parts which form the initiation points of mechanical failure or corrosion. Under this condition, the corrosive medium could readily reach the substrate, and the exposed areas generate local dissolution [18,20]. This reaction further weakens the interface strength between the substrate and the film or coating, resulting in subsequent peels [21]. In this case, different strategies have been employed, such as increasing thickness [22], multilayer design [22], and optimization of the composition [23], to improve the corrosion behavior.

Generally, thicker coatings show better corrosion resistance for decreasing penetrable defects. However, the problem of increasing initial cost to achieve a thicker coating also appears, accompanied with poor adhesion between the coating and copper substrate. With regard to application, a protective coating with a proper thickness that is as low as possible is promising. Multilayer coatings are often used to improve the anticorrosive behavior of different substrates [24]. It has been proven that multilayer coating via the MS method exhibits improved corrosion resistance compared with the corresponding monolayer coating [17,25], as the interface between different layers in the coating plays an important role in the improvement. In a multilayer design, the incorporation of an interlayer between the substrate and coating also helps in achieving good adhesion, with high corrosion resistance. Alloying may enhance corrosion resistance by decreasing the size and density of micro-pores in an MS coating. Silicon is an important alloying element. It has been reported that the incorporation of 16 at.% and 18.4 at.% Si in CrSiN coatings or 1.3 at.% and 1.4 at.% Si in CrSiCN coatings promotes grain refinement and densification of the structure of the coatings, which also enhances corrosion resistance [26]. It has also been reported that the addition of Si to a CrAlN coating disrupts columnar growth and causes the formation of nanocrystalline CrAl(Si)N grains, making it appear smoother without a distinct columnar structure, as observed in CrAlN [27]. In addition, the alloying elements used can generally form a more noble protective layer during the chemical attack of Cl^−^ and O^−^ in sea water [28], as well as those of the corroded products [29]; for example, Cr as an alloying element can easily form a dense Cr_2_O_3_ layer on the coating surface, which passivates the surface and prevents further corrosion attack [22]. Up to now, few investigations have been available on aluminum as an alloying element in MS coatings for application in marine service, although its oxide is very good for protection from further oxygen corrosion.

The purpose of the current study is to provide insight into the protection of copper for use as a heat exchanger in sea water with seriously polluted chloride ions. A Ni/NiCr/NiCrAlSi composite coating was designed for the protection considering both corrosion resistance and thermal conductivity. First, a thick Ni coating was deposited on the copper substrate by electroplating, as it is both an interlayer and an efficient barrier, it has high deposition velocity (low cost) which is very difficult to achieve using PVD techniques, and it has good resistance to sea water and excellent adhesion strength with copper. In addition, for a long time, nickel has been an important element used for corrosion-resistant materials in marine service, which is attributed to the unfilled “d” shell when the nickel content reaches a proper value [30]. A NiCr coating was employed on the Ni coating as both the second interlayer and good corrosion-resistant layer, with high thermal conductivity (171 W/m·K). At last, a NiCrAlSi coating was deposited as the upper layer, considering the potential higher corrosion resistance than the NiCr layer by adding alloying elements of aluminum and silicon. Both the NiCr and the NiCrAlSi coatings were deposited by DC magnetron sputtering. The composite coating and the effect of the content of aluminum in the NiCrAlSi layer were investigated on the structure by scanning electron microscopy (SEM), energy dispersive spectroscopy (EDS), and X-ray diffraction (XRD), while the corrosion behavior was investigated using potentiodynamic polarization and electrochemical impedance spectra (EIS). After that, the corroded morphologies of the composite coating were investigated. Furthermore, the corrosive resistance mechanism of the composite coating, especially the upper part in sea water, is discussed, which has a certain degree of scientific guiding value for its practical application in a marine environment.

## 2. Experimental

### 2.1. Preparation of the Composite Coatings

Copper plates with a dimension of 20 mm × 20 mm × 3 mm were chosen as substrates and were first sequentially polished using 240, 400, 800, 1000, and 1500 SiC grit papers; then, they were sonicated in acetone for 10 min and rinsed with distilled water. After that, a Ni coating about 20 μm in thickness was prepared by electroplating on the pretreated copper substrates under magnetic stirring for 30 min in a Ni-Watts bath (pH = 4). The current density was set at 4 A/dm^2^ and the bath temperature was kept at 50 °C for the deposition. The chemical compositions of the Ni-Watts plating baths are listed in Table 1.

The copper substrates after electroplating Ni were further treated by ultrasonic cleaning in alcohol and acetone solution; then, they were rinsed in deionized water and dried with a blower before being inserted into a vacuum chamber of a magnetron sputtering system (JGP450, SKY Technology Development Co., Ltd., Shenyang, China) for deposition of both the NiCr and the NiCrAlSi layers. Two targets made via powder metallurgy with purities >99.99% in an atom ratio of 80:20 for NiCr and 60:13:5:20 for NiCrAlSi were employed with a diameter size of 60 mm for the sputtering. Contents of aluminum were also designed by putting some extra aluminum powders with a size of 4 mm^3^ on the target surface homogeneously in the NiCrAlSi layer. The distance between the target and substrate was kept at approximately 60 mm. Prior to deposition, the chamber was evacuated to a base pressure of 4.0 × 10^−4^ Pa, and then Ar was introduced for sputtering cleaning to remove the possible contaminants on the substrate at a pulse bias of −800 V and duty cycle of 30% for 5 min. During deposition, the Ar flow rate was kept at 35 sccm, the working pressure was 0.6 Pa, the bias was set as −150 V, which was optimized to ensure good adhesion between the coatings and the substrates, and a DC power of 60 W was chosen for deposition of both the NiCr layer and the NiCrAlSi layer. The deposition durations for the NiCr layer and the NiCrAlSi layer were set to 60 and 30 min, respectively.

### 2.2. Characterization

A morphology examination on the surface and cross-section of the composite coatings were carried out using a S-3400N (Hitachi, Tokyo, Japan) scanning electron microscope (SEM). The samples for the cross-sectional analysis were obtained by being broken after dipping into liquid nitrogen for 15 s. The elemental composition distribution on the surface was determined using a GENESIS 4000 (EDAX, Warrendale, PA, USA) energy dispersive spectroscope (EDS) attached to the SEM. Furthermore, the morphological studies on the composite coating after electrochemical measurements were also carried out using SEM and EDS equipment. X-ray diffraction (XRD) analysis was used to determine the composite coatings by an XRD-7000 (Shimatsu Corporation, Kyoto, Japan) X-ray diffractometer with Cu Ka radiation (wavelength = 0.15406 nm) in a scanning range of 10°–100°. The scanning speed and scanning step were set to 8°/min and 0.02°, respectively. Identification of the phase structure was carried out using the Joint Committee on Powder Diffraction Standards (JCPDS) database.

### 2.3. Electrochemical Measurements

The electrochemical tests were performed on samples with an exposed area of 2 × 2 cm^2^ at room temperature (25 °C ± 1 °C) in a 3.5 wt.% NaCl solution (pH 6.70), which was prepared using sea water from Yingkou city in Liaoning Province, China. Potentiodynamic polarization and electrochemical impedance spectroscopy (EIS) were performed using a CHI604E electrochemical workstation (Chenhua, Shanghai, China) with a standard three-electrode cell, where a platinum (Pt) plate and a saturated calomel electrode (SCE) were used as the counter electrode and reference electrode and the coated specimens were used as the working electrodes, respectively. After the working electrodes were immersed in the 3.5 wt.% NaCl solution until a steady open circuit potential was recorded, the polarization and EIS measurements were carried out depending on the open circuit potential of the working electrodes, with a sinewave disturbance amplitude of ±10 mV and a frequency range from 10^5^ to 10^−2^ Hz. Corrosion current density values, I_corr_, were determined using Tafel extrapolation depending on the potentiodynamic polarization curves. To ensure the reproducibility of the results, the experiments were repeated three times.

## 3. Results and Discussion

### 3.1. Composition and Structure of the Ni/NiCr Composite Coating

**Figure 1:**
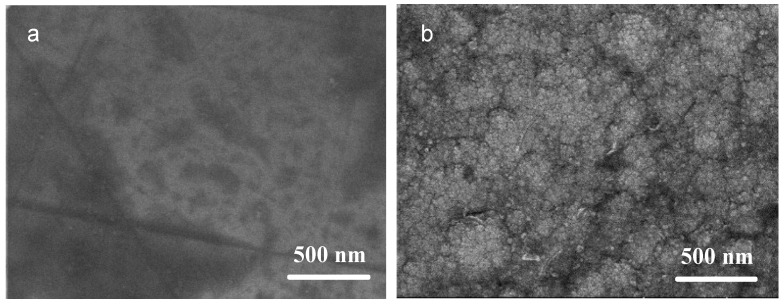
Surface morphologies of (**a**) S1: Ni coating and (**b**) S2: Ni/NiCr coating.

Surface characterization of samples S1 (Ni coating) and S2 (Ni/NiCr coating) was performed. Figure 1 displays the SEM surface morphologies of the two samples at high magnification. Sample S1 exhibits a smooth and dense surface, characteristic of electroplated Ni coatings. In contrast, the surface of sample S2, representing a NiCr coating deposited via sputtering, consists of continuous, compact grain clusters ranging from several to tens of nanometers in size. EDS analysis confirmed the intended composition of the Ni and NiCr coatings, as shown in Table 2. The coating compositions corresponded closely with those of the sputtering target, with minor discrepancies possibly arising from differential sputtering yields of the elements.

**Figure 2:**
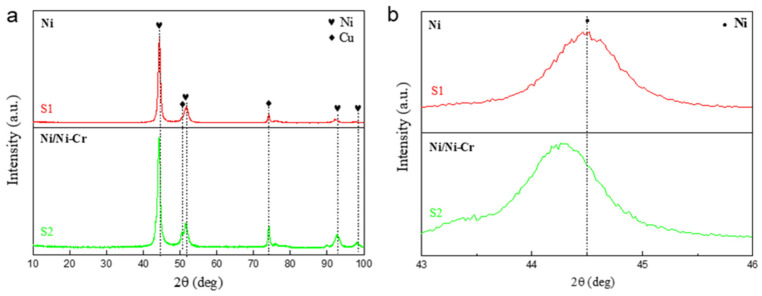
XRD patterns (**a**) and the magnified part (**b**) of the Ni and Ni/Ni-Cr coatings.

X-ray diffraction (XRD) analyses were performed on samples S1 and S2, as depicted in Figure 2, to further investigate their composition and crystal structure. The diffraction patterns revealed peaks solely corresponding to metals Ni and Cu. Analysis using Jade 6 software identified a pronounced peak at 2θ = 44.3°, attributed to the (111) lattice plane of metallic nickel (JPCDS no. 06-0850). Peaks at approximately 50.4° and 74.1° were associated with the copper substrate (JPCDS no. 003-1018). The absence of distinct chromium peaks suggests that Cr does not exist as a separate element in the NiCr layer but likely occupies lattice sites within the Ni matrix as an alloying component. Notably, the sharp (111) plane peak for sample S2 shifts to a lower diffraction angle, indicative of the larger atomic radius of the alloying Cr element. Given that the atomic radii of alloying atoms Cr, Al, and Si are 0.127 nm, 0.143 nm, and 0.134 nm, respectively, all larger than Ni’s radius of 0.124 nm, this disparity induces lattice distortion and increases interplanar spacing in the Ni matrix. Consequently, the diffraction peak shifts to a smaller angle due to the Bragg equation (2d_hkl_sinθ = nλ, where d represents the interplanar distance and θ is the Bragg angle). These findings corroborate the hypothesis that Cr replaces Ni and solubilizes within the Ni lattice in the NiCr layer.

### 3.2. Effect of Aluminum Content in NiCrAlSi Layer on the Structure and Properties

**Figure 3:**
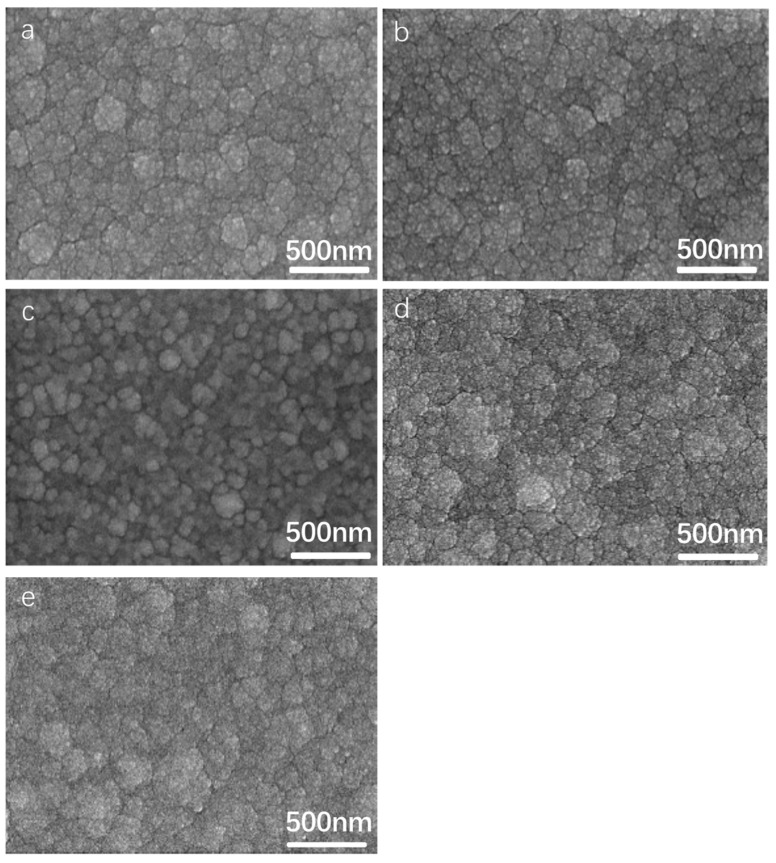
Surface morphologies of the Ni/NiCr/NiCrAlSi composite coatings with different Al contents. (**a**) B1-4.3 at.%, (**b**) B2-5.49 at.%, (**c**) B3-6.98 at.%, (**d**) B4-8.06 at.%, and (**e**) B5-9.93 at.%.

The NiCrAlSi coating was deposited as the upper layer for its anticipated enhanced corrosion resistance. Given the potential impact of aluminum content in the NiCrAlSi layer on the corrosion resistance of the protective coating, a series of Ni/NiCr/NiCrAlSi coatings with varying Al contents were developed and applied to copper substrates, which was achieved by adjusting the quantity of aluminum grains on the sputtering target surface. This study aimed to explore the influence of the Al content on the composition, structure, and electrochemical behavior of the composite coating. The SEM surface morphologies of Ni/NiCr/NiCrAlSi coatings with different Al contents are illustrated in Figure 3. The analysis reveals that the NiCrAlSi layers consist of continuous and dense grain clusters, with sizes varying from a few to tens of nanometers. Among the samples, B2, which contains 5.49 at.% Al, has the smallest average grain size, which is attributed to silicon (Si)-induced grain refinement [26].

The EDS results in Table 3 validate the successful deposition of NiCrAlSi layers with varying aluminum (Al) contents, categorizing the samples from B1 to B5. These results reveal that the Si content in the NiCrAlSi layer, as well as its proportion relative to the combined content of Ni and Cr, is considerably higher in samples B1 and B2, with B2 having the highest percentage. It is suggested that higher Si content enhances grain refinement, leading to a reduced average grain size in NiCrAlSi coatings. Notably, the Al concentration in the coatings increases proportionally with the number of aluminum grains on the target surface, while the levels of Ni and Cr remain relatively constant throughout the process. However, the Si content and its ratio to the combined Ni and Cr content initially increase and then sharply decrease. It is hypothesized that NiCrAlSi coatings with a greater proportion of smaller grains or grain boundaries will demonstrate improved corrosion resistance. This is attributed to corrosion products such as Cr_2_O_3_, Al_2_O_3_, and SiO_2_ films, which are more noble and act as barriers to corrosive solutions. Further investigation and discussion of this hypothesis will be presented in a subsequent paper.

**Figure 4:**
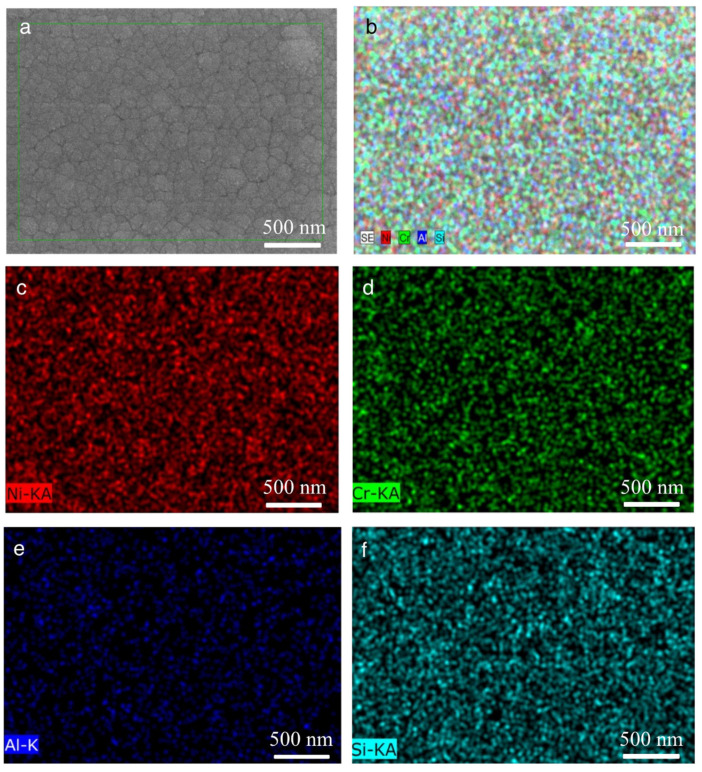
SEM-EDS map scan on surface of Ni/NiCr/NiCrAlSi composite coating with Al content at 5.49 at.%. (**a**) Surface image, with the green box indicating the EDS mapping area; (**b**) color map; (**c**) Ni distribution; (**d**) Cr distribution; (**e**) Al distribution; (**f**) Si distribution.

As magnetron sputtering is a process with a very fast cooling rate, which restricts diffusion when the atoms Ni, Cr, Al, and Si (or their clusters) reach the substrate, the nucleation and growth of intermetallic compounds are restrained, resulting in the formation of a solid solution layer, as intended. Consequently, this process yields a coating with a dense microstructure and a precise chemical composition. Furthermore, the EDS mapping result reveals a uniform distribution of Ni, Cr, Al, and Si within the NiCrAlSi layer, as illustrated in Figure 4, as was the case with Al content at 5.49 at.%.

**Figure 5:**
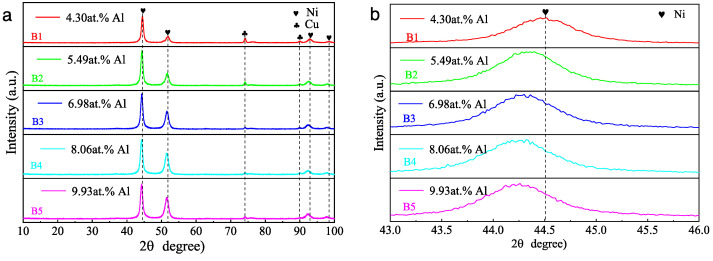
XRD pattern (**a**) and an enlarged part (**b**) of the Ni/NiCr/NiCrAlSi composite coatings with different Al contents.

**Figure 6:**
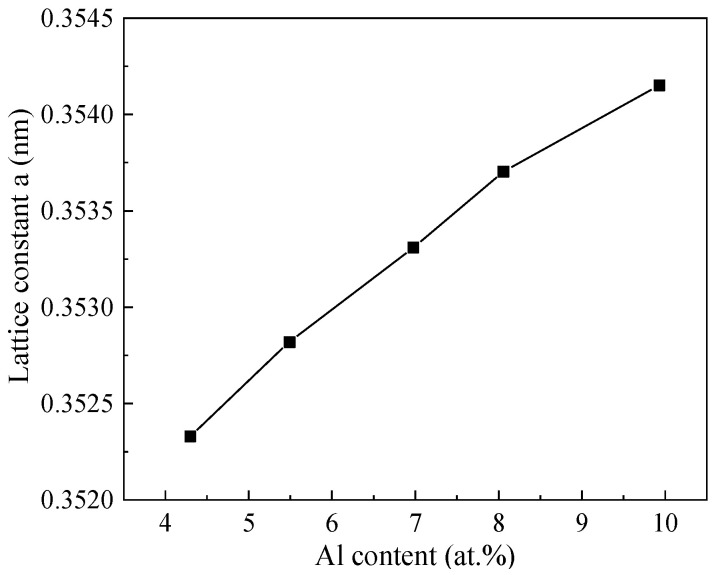
The relationship between lattice constant and Al content for the Ni/NiCr/NiCrAlSi coating.

Figure 5 shows the XRD pattern of the Ni/NiCr/NiCrAlSi composite coatings. Still, only peaks of Ni and Cu are found in the XRD patterns (Figure 5), while no peaks from elements Cr, Al, and Si appear on the Ni/NiCr/NiCrAlSi coatings with different Al contents. Analyzed with the software, the peaks centered at 2θ of about 44.4°, 51.5°, 92.5°, and 98.1° (with the sharp and intensive peaks located at 2θ = 44.4°) are assigned to the 111, 220, 311, and 222 diffraction lattice planes of Ni, which are 44.5°, 51.8°, 92.9°, and 98.4° for Ni in the JCPDS database (JPCDS no. 06-0850), while those at 74.1° and 89.9° correspond to the Cu substrate (JPCDS no. 003-1018). It is clear that there is a negative shift in the diffraction angle for the composite coating with the Al content from that of the matrix Ni, and the higher the Al content is, the more the negative shift. According to the Bragg equation and the equation for the lattice constant (d_hkl_ = a/(h^2^ + k^2^ + l^2^)^1/2^, where a is the lattice constant and h, k, and l are the diffraction index), the lattice constants of the NiCrAlSi layer with different Al contents are calculated and shown in Figure 6. It is indicated that the lattice constant of the NiCrAlSi layer increases with the increasing Al content in the layer, and the higher the Al content is, the more the lattice distortion (r_Al_ = 0.143 nm, r_Ni_ = 0.124 nm) appears, resulting in a longer distance and negative shift on diffraction angle in turn, which proves once more that Al takes the place of Ni, not as an interstitial atom in the lattice. It should be mentioned that there is only a little negative shift on the peak at 44.4° on the sample with Al content at 4.30 at.%, indicating that the Al content should be higher than this value for an apparent lattice distortion in the NiCrAlSi layer.

**Figure 7:**
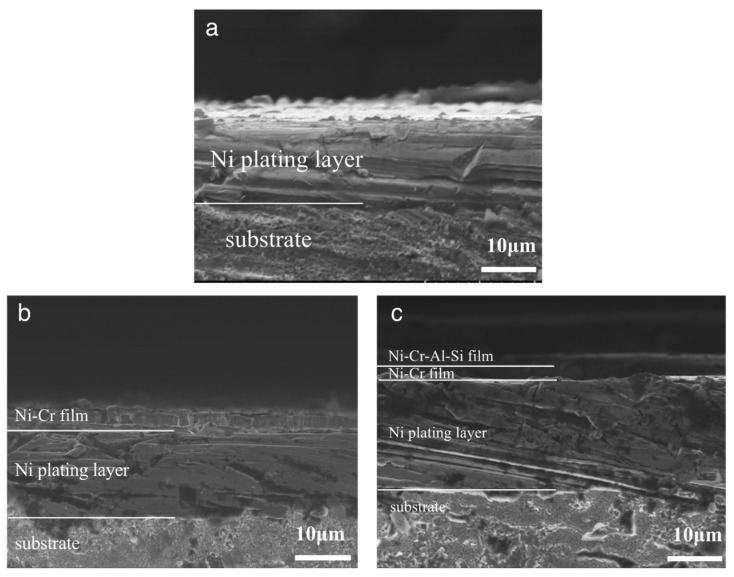
Cross-sectional morphologies of the Ni (**a**), Ni/NiCr (**b**), and Ni/NiCr/NiCrAlSi (**c**) coatings.

Figure 7 shows the SEM images of the three protective coatings on a cross-section. It is demonstrated that the composite coatings (Ni/NiCr and Ni/NiCr/NiCrAlSi) are composed of two layers and three layers, respectively, as intended. All three of the coatings adhere well to the substrate and no defects such as pores or holes are found at the interface. The thickness of the three coatings is about 20 μm, although it is hard to distinguish the interface between the different layers in the cases of the Ni/NiCr and Ni/NiCr/NiCrAlSi coatings. Furthermore, a distinct columnar crystal structure can be found on both the NiCr and the NiCrAlSi layers, which is a typical characteristic of coatings formed through magnetron sputtering. It is accepted that there is good adhesion between electroplated Ni and Cu substrates and that they have excellent affinity. Furthermore, there is an element gradient from the Ni layer to the NiCr layer until the NiCrAlSi layer, which is predicted to be helpful in terms of relief on internal stress at the interface. The flat interfaces in Figure 7 confirm good adhesion and compatibility between each layer.

**Figure 8:**
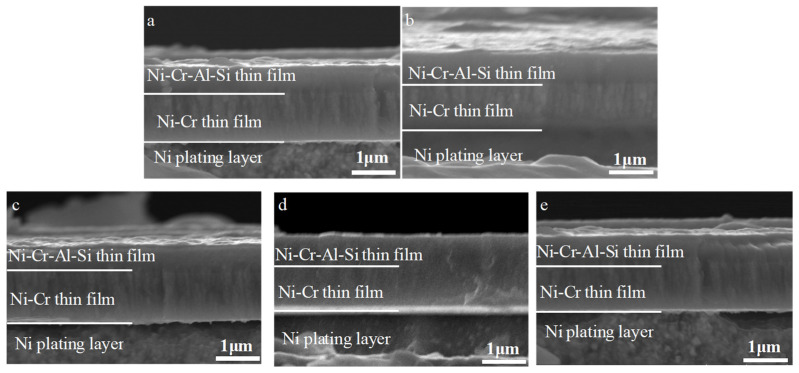
Cross-sectional morphologies of the NiCr/NiCrAlSi layers with different Al contents. (**a**) B1-4.3 at.%, (**b**) B2-5.49 at.%, (**c**) B3-6.98 at.%, (**d**) B4-8.06 at.%, and (**e**) B5-9.93 at.%.

There is not much difference in the cross-section images for the Ni/NiCr/NiCrAlSi coatings with different Al contents, as shown in Figure 8. All of the coatings adhered well and no defects such as pores or holes were found at the interface between the NiCr layer and the NiCrAlSi layer. The thickness of the NiCr layer in the Ni/NiCr/NiCrAlSi coatings was about 2 μm, while the thickness of the NiCrAlSi layer was about 1 μm. Furthermore, a distinct columnar crystal structure could be found more clearly on both the NiCr layer and the NiCrAlSi layer when using higher magnification.

### 3.3. Corrosion Behavior of the Ni/NiCr Coating

#### 3.3.1. Potentiodynamic Polarization Measurements

**Figure 9:**
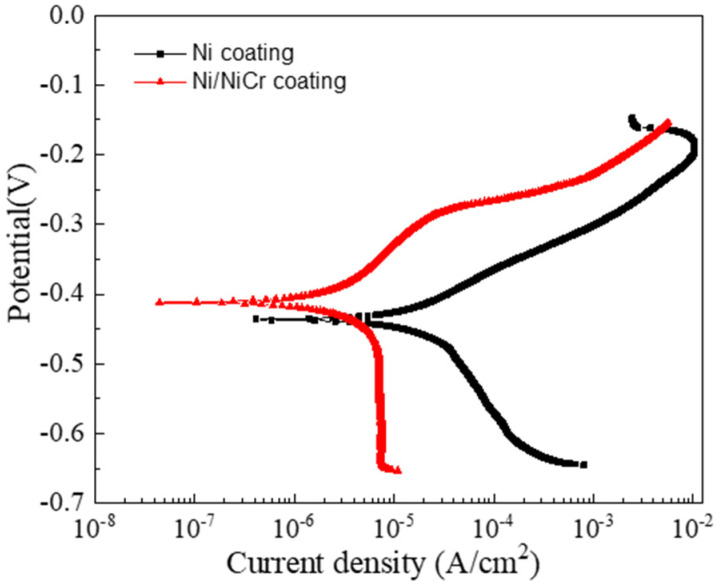
Potentiodynamic polarization curves of the Ni nd Ni/NiCr coatings.

The polarization curves for the Ni and Ni/NiCr coatings on the Cu substrate are shown in Figure 9. By using the Tafel technique based on the polarization curves, the corrosion potential E_corr_ is obtained, which is about −0.437 V and −0.412 V for the Ni and the Ni/NiCr, respectively. It is clear that the E_corr_ values shift to a positive direction after the NiCr coating deposition. The corrosion current density exhibited a notable decrease from 2.304 × 10^−5^ A/cm^2^ to 6.551 × 10^−6^ A/cm^2^ following the application of the NiCr coating, indicating improved corrosion resistance. It is known that Ni and Cr are more prone than Cu to chemical attacks. However, the growth defects (pinholes, inter-columnar voids, grain boundaries, etc.) during the deposition may allow corrosive media to enter the coatings, thus degrading the corrosion resistance. The result that the Ni/NiCr coating presents better corrosion resistance stems from the interface between the two layers and the chemical compositions of the upper layer, omitting the effect of the coating thickness. It is accepted that the interfaces of multilayer coatings can impede the growth of defects and cut off the corrosive medium path to the substrate [22]. So, the corrosion resistance of the Ni/NiCr coating is improved more than that of the Ni coating.

#### 3.3.2. AC Impedance Measurements

**Figure 10:**
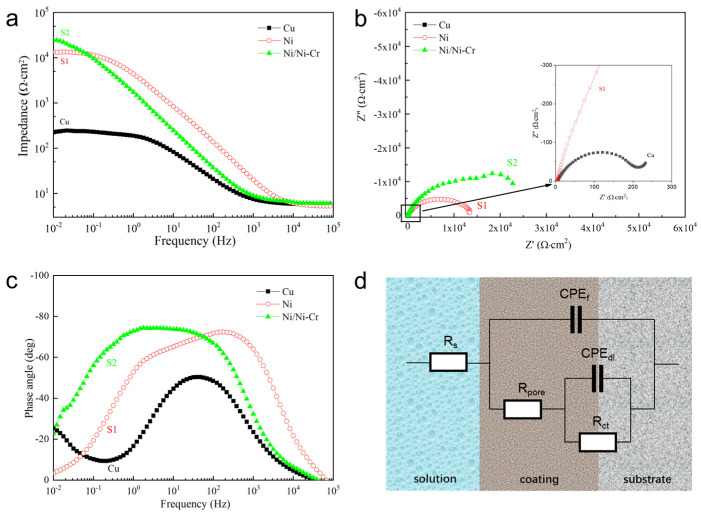
Bode impedance (**a**), Nyquist (**b**), and Bode phase angle (**c**) plots of the Ni and Ni/NiCr coatings using Cu as a reference, and their equivalent circuit (**d**) to simulate the corrosion reaction.

Impedance spectra of the Ni and Ni/NiCr coatings are compared in Figure 10a, using Cu as a reference. As it is accepted that the impedance value at a low frequency reflects the corrosion resistance of a sample, the bigger the impedance is, the higher the corrosion resistance is. Between the two protective coatings, the bigger impedance modulus at a low frequency is found on the Ni/NiCr coating, which agrees with the results of the potentiodynamic polarization measurement and proves that the Ni/NiCr coating possesses higher corrosion resistance. The Nyquist diagrams (Figure 10b) show a presence of semicircles with different radii for the two coatings. In the case of copper substrate, a small semicircle can also be found in a magnified figure inserted in the right hand corner. It is indicated that the semicircle radii of both of the two protective coatings are much bigger than that of the Cu substrate. The semicircle radius is bigger for the Ni/NiCr coating, which agrees with the results in the AC impedance measurement in Figure 10a. In general, the bigger the semicircle radius is, the higher the corrosion resistance of the protective coating is, indicating greater resistance for charge transfer during the corrosion process. The same tendency on Bode spectra appears on the two protective coatings, as shown in Figure 10c. It can be found that two obvious time constants on the Bode spectra appear for the Ni and Ni/NiCr coatings, while only a time constant appears for the Cu substrate. It is understood that there are microholes on the surface of the electroplated Ni coating, and the corrosion solution can penetrate into the Cu substrate more easily, so the corrosion microbattery is formed in the interface area, and the impedance spectrum will show two obvious time constants. In the case of the Ni/NiCr coating, the NiCr layer on the Ni coating was prepared by magnetron sputtering, which is relatively dense, resulting in it being more difficult for the electrolyte solution to penetrate into the interface. It can be seen from the Bode spectra that the phase angle increases following Cu, Ni, and Ni/NiCr, and that of the Ni/NiCr composite coating is as low as ~−77° in a wide frequency range. It is generally believed that the greater the absolute value of the maximum phase angle, the better the electrochemical corrosion resistance is. Therefore, the corrosion resistance of the Ni/NiCr composite coating is better than that of the Ni coating, which is also in agreement with the results in the Bode impedance spectra (Figure 10a) and Nyquist diagram (Figure 10b).

The equivalent circuit for the protective coatings is shown in Figure 10d, where R_s_ is the solution resistance; R_pore_ is the resistance of defects and pores in the coating; R_ct_ is the charge transfer resistance of the electrolyte/substrate interface; and CPE_f_ and CPE_dl_ are the double-layer capacitance of the coating/electrolyte and substrate/electrolyte interface, respectively. The parameters obtained by fitting the equivalent circuit using ZsimpWin 3.30d software are shown in Table 4. It can be found that the R_pore_ and R_ct_ vary with the protective coatings. The Ni/NiCr coating exhibits much higher values on R_pore_ and R_ct_ than those of the Ni coating, which indicates that the interface between different layers or between the coating and the substrate acts to cut off the corrosive medium path to the substrate. The coatings with more layers possess more interfaces, and thus present higher resistance (R_pore_) in coating pores as the interface greatly reduces the length of the pores and decreases the quantity of the penetrable pinholes to reach the substrate. The R_ct_ increases in value following Ni coating and Ni/NiCr coating. The results can be explained in two aspects. The higher R_ct_ of the Ni/NiCr coatings is attributed to both the smaller contact area between the electrolyte and surface in coating pores, and a more difficult charge transfer process in the electrolyte/NiCr interface due to alloying the element Cr.

#### 3.3.3. SEM Observation on Sample Surface after Potentiodynamic Polarization Measurement

**Figure 11:**
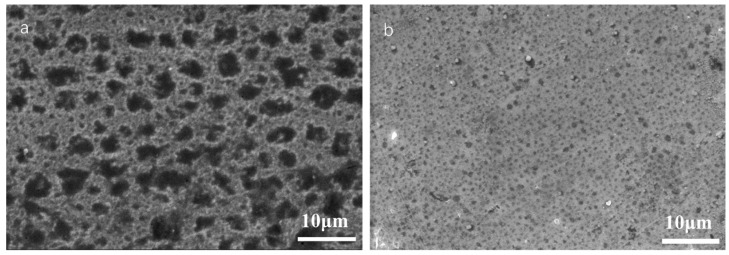
Surface morphologies of the Ni (**a**) and Ni/NiCr (**b**) coatings after potentiodynamic polarization measurements.

The surface morphologies of the samples after potentiodynamic polarization measurements were observed and are shown in Figure 11. Pitting corrosion occurs on the surface, and a significant difference is found on the protective coatings. There are many holes with widths of several micrometers on the surface of the Ni coating sample. It seems the holes with sizes of several to ten micrometers are merged by several smaller holes, as some of them are also found on the surface. In the case of the Ni/NiCr coating sample, although there are still many holes on the surface, the average size of the holes decreases to about 1 μm. Evidently, pitting is the corrosion mechanism for the protective coatings. It is predicted that pits are formed in some pores or other growing defects on the coating surface as a result of a corrosion attack. The pits are mainly a result of the full corrosion or preferential dissolution of some elements or compositions, which finally form holes. It is indicated that there are many growing defects in the electroplated Ni coating although it is dense from the surface image. On the basis of protection by the Ni coating, almost the same number but much smaller holes appear on the surface of the NiCr layer, which may stem from both the denser structure by magnetron sputtering and the alloying element Cr, another noble element.

In general, when the Ni or Ni-based coating samples are immersed in an electrolyte, the corrosion is expected to rapidly initiate not only on the pores but also on other growing defects in the coatings. This leads to the formation of localized galvanic cells, which dominate the galvanic corrosion process. In such cases, the electrochemical interface can mainly be divided into three sub-interfaces, such as electrolyte/coating, electrolyte/defect, and electrolyte/substrate. The corrosion of defects finally forms holes. Actually, the interface presents double actions in a corrosion process. The interfaces in the coatings may block the micro-pores and cut off the corrosive medium path to the substrate. On the other hand, they can also accelerate the corrosion process because a galvanic corrosion process also appears between the interface and matrix. The effect of the interface in a coating on the corrosion resistance may depend on the degree or ratio of the two effects.

### 3.4. Corrosion Behavior of the Ni/NiCr/NiCrAlSi Coating

**Figure 12:**
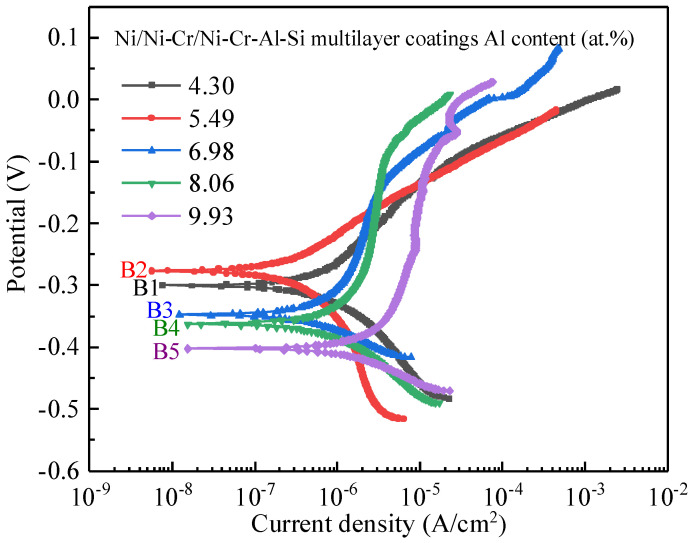
Potentiodynamic polarization curves of the Ni/NiCr/NiCrAlSi coatings with different Al contents.

Figure 12 shows the potentiodynamic polarization curves of the five samples covered by Ni/NiCr/NiCrAlSi coatings with various Al contents in the upper layer. The curves are divided into three regions: Region I corresponds to the polarization region in the vicinity of the OCP; Region II, at the more negative potentials, is ascribed to oxygen reduction under mass transfer control; and Region III, rising at the negative potentials between −0.3 V and −0.5 V vs. SCE, is unambiguously attributed to the hydrogen evolution reaction. As shown in Table 5, it is clear that the corrosion potentials of the composite coatings increase first, reach the highest value on sample B2, and then decrease with increasing Al content, which is −0.301 V, −0.277 V, −0.348 V, −0.363 V, and −0.403 V on samples B1, B2, B3, B4, and B5, respectively. Among all samples, B2 shows the smallest *i_corr_* of 2.666 × 10^−7^ A·cm^−2^, which is approximately two orders of magnitude lower than that of the Ni coating (S1) and more than one order of magnitude lower than the Ni/NiCr coating (S2). It is therefore inferred that the corrosion resistance of the composite coating is significantly enhanced by the NiCrAlSi layer. More positive *E_corr_* and lower *i_corr_* are generally indicative of greater corrosion resistance [31]. The observed variations in *E_corr_* and *i_corr_* across the samples provide insights into the corrosion dynamics of the coatings. The progression of *E_cor_* values across samples B1 to B5 suggests a strong dependence on Al content within the NiCrAlSi layer. The peak performance in sample B2, which exhibits the most positive *E_corr_* and the lowest *i_corr_*, underscores the optimal balance achieved in this sample’s composition. It is also indicated that sample B2 possesses the highest corrosion resistance, while sample B5 has the lowest corrosion resistance. It seems that there is a relationship between the grain size and the corrosion resistance for the NiCrAlSi layer, and the sample with the smallest grain size for the NiCrAlSi layer (sample B2) corresponds the highest corrosion resistance, which verifies our assumption that corrosion products of Cr, Al, and Si will block the passage of corrosive solution after being immersed into the solution.

**Figure 13:**
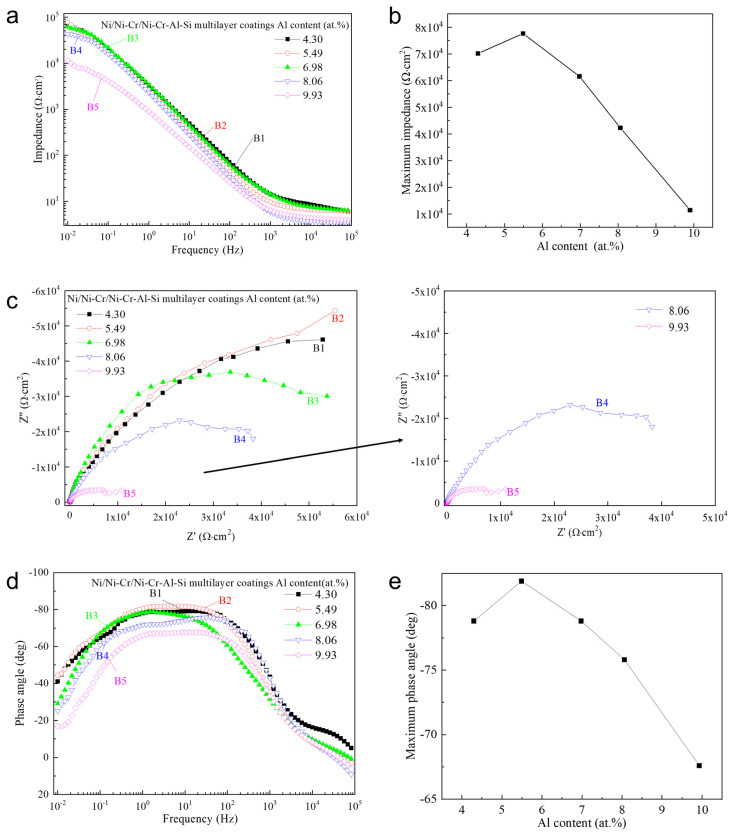
Bode impedance (**a**), the maximum impedance (**b**), Nyquist (**c**), Bode phase angle (**d**), and the maximum phase angle (**e**) plots of the Ni/NiCr/NiCrAlSi coatings with different Al contents.

The AC electrochemical impedance spectroscopy of the Ni/NiCr/NiCrAlSi coatings with different Al contents is shown in Figure 13a. The impedance of the five samples diminishes with increasing frequency and stabilizes beyond 10^3^ Hz, with the greatest impedance modulus observed at the initial frequency. Among the samples, B2 exhibits the highest impedance modulus, correlating with its superior corrosion resistance as indicated by potentiodynamic polarization curves. Notably, sample B5 shows a lower impedance modulus across all frequencies, suggesting that optimal Al content is crucial for enhanced corrosion resistance. An analysis of the data in Table 6 suggests that the AC impedance is influenced by the silicon to nickel + chromium ratio ([Si]/[Ni + Cr]), with a higher ratio yielding greater impedance. This trend underscores silicon’s significant role in the corrosion resistance of the Ni/NiCr/NiCrAlSi composite coating. The beneficial effect of silicon is twofold: it impedes the growth of the NiCrAlSi solid solution within the nickel matrix, increasing with the Si to Ni + Cr ratio, leading to a finer grain size, a denser surface, and consequently, heightened corrosion resistance. Additionally, the formation of a silicon oxide film post-immersion in the electrochemical testing solution, which acts as a barrier to further corrosion and electric charge transfer, further contributes to the coating’s corrosion resistance.

The Nyquist diagrams (Figure 13b) show that the radii of semicircles are connected with the Al content in the NiCrAlSi layer, which increases at first and reaches the highest value on sample B2 and then decreases with the increasing Al content. In the case of sample B5, the radius is so small that it is extinguished by an enlarged image inserted in the right column. The same tendency appears on the Bode spectra on the Ni/NiCr/NiCrAlSi composite coating with different Al contents, as shown in Figure 13c. The same relationship appears for the five samples, where the biggest impedance modulus is between the maximum phase angle and the Al content. For sample B2 (aluminum content = 5.49 at.%), the biggest phase angle reaches −85°.

The equivalent circuit parameters, fitted to the equivalent circuit using ZsimpWin software for the five samples with varying Al contents, are presented in Table 6. Analysis reveals that sample B2 has the largest charge transfer resistance (R_ct_), suggesting the most substantial barrier to charge transfer at the electrolyte/coating interface. In this study, the optimal aluminum (Al) content was determined to be 5.49%, which yielded the highest charge transfer resistance (R_ct_), maximum impedance modulus, and largest phase angle, aligning with the enhanced corrosion resistance observed in polarization tests. This optimal concentration of Al not only improves the overall structural integrity of the film but also enhances its corrosion resistance capabilities. The presence of Al in the alloy composition plays a crucial role in the formation of a protective film on the coating surface. The increase in R_ct_ for sample B2 suggests that this specific Al content effectively contributes to a more robust barrier against charge transfer, which is a key factor in corrosion resistance. The enhanced impedance properties, as observed in the EIS data, further support this conclusion, indicating more effective resistance to the electrochemical processes that drive corrosion. However, the diminishing performance with higher Al content beyond this optimal point highlights the complex nature of alloying in corrosion resistance. The more reactive nature of Al, as compared to Cr and Ni, suggests that excessive Al could lead to destabilization of the passivation film. This phenomenon can be attributed to the inherent electrochemical properties of Al, which, despite contributing to passivation, may also induce instability in the protective layer in aggressive environments. The trend observed in the EIS data, where increasing Al content beyond a certain threshold leads to decreased corrosion resistance, underscores the need for a balanced alloying approach. It suggests that while Al is beneficial up to a certain concentration, its excess can counteract the benefits and lead to a reduction in protective properties.

**Figure 14:**
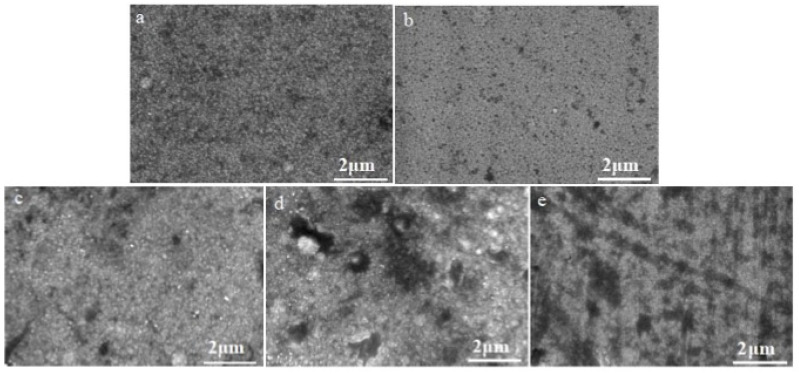
Surface morphologies of the Ni/NiCr/NiCrAlSi coatings with different Al contents after potentiodynamic polarization measurement. (**a**) B1-4.3 at.%, (**b**) B2-5.49 at.%, (**c**) B3-6.98 at.%, (**d**) B4-8.06 at.%, and (**e**) B5-9.93 at.%.

After potentiodynamic polarization measurements, surfaces were observed and compared for all five samples, as shown in Figure 14. Pitting corrosion occurs on all sample surfaces. However, only several smaller holes appear on the surface of the NiCrAlSi layer, indicating that the new alloying elements Al and Si possibly play an important role in the further improvement in corrosion resistance, which will be discussed in detail in the following section. In the case of sample B4, large pits with sizes of about 1–2 μm are found. However, there are only several small pits on the surface of sample B2 with sizes of about several tens to a hundred nanometers, and those of sample B1 are about several hundred nanometers. This result proves once more that sample B2 possesses the best corrosion resistance, and implies that the Al content in the NiCrAlSi layer should be in a proper range for an improvement in corrosion resistance for the Ni/NiCr/NiCrAlSi composite coating.

## 4. Conclusions

This research successfully developed and analyzed a Ni/NiCr/NiCrAlSi composite coating to enhance the corrosion resistance of copper, particularly for its application in marine heat exchangers. This study employed comprehensive characterization techniques, including SEM, EDS, XRD, potentiodynamic polarization, and EIS, to elucidate the composition, structure, and corrosion behavior of the coating in a marine environment. The multilayer structure, particularly the upper NiCrAlSi layer, plays a crucial role in this enhanced resistance, with the aluminum content being a critical factor. This study demonstrates that pitting corrosion, primarily in NaCl solutions, is effectively mitigated by this composite coating. Furthermore, the research provides valuable insights into the optimization of aluminum content within the NiCrAlSi layer, underlining its importance in achieving the ideal balance between corrosion resistance and thermal conductivity. The results highlight that a specific range of aluminum content maximizes corrosion resistance, as evidenced by electrochemical testing and surface analysis post-corrosion.

In conclusion, the Ni/NiCr/NiCrAlSi composite coating presents a robust and efficient solution for protecting copper in marine applications, offering significant advancements over traditional methods. Its superior corrosion resistance, coupled with retained thermal conductivity, makes it an excellent candidate for use in heat exchangers in mariculture and other marine environments. Future research could explore the further optimization of this coating, potentially broadening its application scope within various industrial sectors.

## Figures and Tables

**Table 1:** The bath chemical compositions used for electroplating Ni.

Component	Concentration (g/L)
NiSO_4_·6H_2_O	200
NiCl_2_	15
H_3_BO_3_	41
Additive (Coumarin)	3

**Table 2:** Elemental atomic concentration (at.%) of S1, S2, and the target used for NiCr layer deposition.

Sample	Ni (at.%)	Cr (at.%)	Al (at.%)	Si (at.%)
S1	100	0	0	0
S2	81.43	18.57	0	0
Target for NiCr deposition	80	20	-	-

**Table 3:** Elemental atomic concentration (at.%) of Ni/NiCr/NiCrAlSi composite coatings with different Al contents and the target used for NiCrAlSi deposition.

Sample	Ni (at.%)	Cr (at.%)	Al (at.%)	Si (at.%)	[Si]/[Ni + Cr]
B1	63.51	12.50	4.30	19.69	0.259
B2	61.80	12.36	5.49	20.35	0.274
B3	62.57	12.57	6.98	17.88	0.237
B4	62.07	12.54	8.06	17.33	0.232
B5	61.26	12.33	9.93	16.49	0.224
Target	60	13	5	20	0.274

**Table 4:** EIS data corresponding to equivalent circuit and standard parameters of S1 and S2 coatings.

Sample	*R_s_/*Ω·cm^−2^	*CPE_f_/*μF·cm^−2^	*n_c_*	*R_pore_/*Ω·cm^−2^	*CPE_dl_/*μF·cm^−2^	*n_t_*	*R_ct_/*Ω·cm^−2^
S1	5.279	2.121 × 10^−5^	0.8972	982.9	3.014 × 10^−5^	0.6964	1.299 × 10^4^
S2	6.212	1.216 × 10^−5^	0.8459	2.029 × 10^4^	2.283 × 10^−5^	0.5033	2.55 × 10^4^

**Table 5:** *E_corr_*, *i_corr_*, and *β_c_* of Ni/NiCr/NiCrAlSi coatings with different Al contents in 3.5 wt.% NaCl calculated from the polarization curves.

Sample	*E_corr_* (V Vs. SCE)	*i_corr_* (A·cm^−2^)	*β_c_* (V/Decade)
B1	−0.301 ± 0.013	(5.461 ± 0.195) × 10^−7^	−0.127 ± 0.006
B2	−0.277 ± 0.022	(2.666 ± 0.171) × 10^−7^	−0.132 ± 0.009
B3	−0.348 ± 0.011	(7.299 ± 0.32) × 10^−7^	−0.080 ± 0.008
B4	−0.363 ± 0.012	(1.046 ± 0.043) × 10^−6^	−0.108 ± 0.011
B5	−0.403 ± 0.023	(1.567 ± 0.093) × 10^−6^	−0.070 ± 0.007

**Table 6:** EIS data corresponding to equivalent circuit and standard parameters of the Ni/NiCr/NiCrAlSi coatings with different Al contents.

Sample	*R_s_/*Ω cm^−2^	*CPE_f_/*μF·cm^−2^	*n_c_*	*R_pore_/*Ω cm^−2^	*CPE_dl_*/μF·cm^−2^	*n_t_*	*R_ct_/*Ω cm^−2^
B1	5.577	5.502 × 10^−5^	0.7369	7.445 × 10^4^	1.69 × 10^−5^	0.9614	1.445 × 10^5^
B2	6.469	7.357 × 10^−5^	0.8906	5.101 × 10^4^	5.97 × 10^−5^	0.6616	1.502 × 10^5^
B3	7.006	5.456 × 10^−5^	0.8329	4.206 × 10^4^	1.015 × 10^−5^	0.9889	8.792 × 10^4^
B4	3.602	8.083 × 10^−5^	0.9128	1.148 × 10^4^	2.173 × 10^−5^	0.4612	4.977 × 10^4^
B5	4.146	2.069 × 10^−5^	1	1686	2.489 × 10^−5^	0.7436	1.108 × 10^4^

## Data Availability

Data are contained within the article.
